# The Influence of Solid Microneedles on the Transdermal Delivery of Selected Antiepileptic Drugs

**DOI:** 10.3390/pharmaceutics8040033

**Published:** 2016-11-15

**Authors:** Julia Nguyen, Kevin B. Ita, Matthew J. Morra, Inna E. Popova

**Affiliations:** 1 College of Pharmacy, Touro University California, Mare Island-Vallejo, CA 94592, USA; julia.nguyen@tu.edu; 2 Department of Plant, Soil and Entomological Sciences, University of Idaho, Moscow, ID 83844, USA; mmorra@uidaho.edu (M.J.M.); ipopova@uidaho.edu (I.E.P.)

**Keywords:** epilepsy, carbamazepine, tiagabine hydrochloride, transdermal drug delivery, microneedles

## Abstract

The aim of this project was to examine the effect of microneedle rollers on the percutaneous penetration of tiagabine hydrochloride and carbamazepine across porcine skin in vitro. Liquid chromatography-mass spectrometric analysis was carried out using an Agilent 1200 Series HPLC system coupled to an Agilent G1969A TOF-MS system. Transdermal flux values of the drugs were determined from the steady-state portion of the cumulative amount versus time curves. Following twelve hours of microneedle roller application, there was a 6.74-fold increase in the percutaneous penetration of tiagabine hydrochloride (86.42 ± 25.66 µg/cm^2^/h) compared to passive delivery (12.83 ± 6.30 µg/cm^2^/h). For carbamazepine in 20% ethanol, passive transdermal flux of 7.85 ± 0.60 µg/cm^2^/h was observed compared to 10.85 ± 0.11 µg/cm^2^/h after microneedle treatment. Carbamazepine reconstituted in 30% ethanol resulted in only a 1.19-fold increase in drug permeation across porcine skin (36.73 ± 1.83 µg/cm^2^/h versus 30.74 ± 1.32 µg/cm^2^/h). Differences in flux values of untreated and microneedle-treated porcine skin using solid microneedles for the transdermal delivery of tiagabine were statistically significant. Although there were 1.38- and 1.19-fold increases in transdermal flux values of carbamazepine when applied as 20% and 30% ethanol solutions across microneedle-treated porcine skin, respectively, the increases were not statistically significant.

## 1. Introduction

The transdermal route of drug administration is advantageous because it avoids shortcomings of oral drug delivery [1]. The advantages of the percutaneous route include painlessness, avoidance of first pass metabolism and the absence of erratic absorption, control over the rate of drug release, easy termination of therapy, and the possibility of self-administration and sustained drug release with a single application [2,3,4]. Despite the obvious advantages of this route of drug administration, only about 20 active pharmaceutical ingredients are used in clinical practice in the form of transdermal patches [5,6,7]. This is because the skin forms a remarkably strong barrier against drug penetration [8]. Percutaneous transport of drug molecules across the skin is challenging because of the structure and components of the skin [8]. The human skin protects the internal organs and permits extremely low drug absorption from the skin surface.

The stratum corneum (SC) hinders transdermal drug delivery [9]. Although 10 to 25 layers of corneocytes provide the physical barrier, corneodesmosomes hold the corneocytes together in an equimolar ratio mixture of ceramides, cholesterol, and free fatty acids [9,10,11]. Percutaneous drug penetration is achieved by either diffusion through the epidermis or through the skin appendages (i.e,. sweat glands and hair follicles) [9]. In healthy skin, lipid composition plays an important role in permeability due to its unique organization, where lipid sheets are stacked parallel to the skin surface [11]. Several techniques are available for enhancing drug absorption through the skin. These include iontophoresis [12], sonophoresis [13], chemical penetration enhancers [14] and microneedles [15].

Microneedles are micron-sized needles with lengths ranging from 100 up to 1000 μm. These devices are capable of facilitating the delivery of drugs across the skin by creating micron-sized pores through the stratum corneum without causing pain or bleeding [16]. The unique structure and geometry of microneedles influence the rate at which drugs diffuse through the skin. These factors include needle length, density, shape, and size in addition to the number of applications and the concentration of drug used [16,17]. The needles must be strong enough to penetrate the tough skin barrier while withstanding the pressure required during insertion [18]. Microneedles are especially important for patients with needle phobias [19,20]. Although microneedle technology has been available for a number of years, it is increasingly being used for the delivery of new compounds and the products of the biotechnological industry. It has been shown that microneedles can be used to deliver proteins, peptides and small interfering RNAs (siRNAs). MNs are minimally invasive and can be administered without damaging nerve endings in the skin [21]. The use of MNs is preferred because these devices are inexpensive and offer more reliable mode of administration by improving the pharmacokinetic and pharmacodynamics profiles of drugs [21]. MNs induce faster healing at administration site compared to hypodermic needles while allowing for a controlled rate of drug delivery [22]. Passive approaches, such as penetration enhancers, result in skin irritation and are limited to only small molecules [6,21]. Other active methods, including electroporation, iontophoresis, magnetophoresis, and sonophoresis, generally require expensive and complex systems [21].

Microneedle rollers continue to attract research interest [23,24]. It is a simple and cost-effective technique to increase transdermal delivery of medications applied to the skin [25]. Microneedle rollers contain micron-sized solid needles that are symmetrically aligned on a cylindrical surface, allowing a rolling movement as it is pushed along the skin surface [17,23,25,26].

Epilepsy is a chronic neurological disorder in which patients experience repeated seizures or excessive amounts of electrical discharges in brain cells [27]. This disorder involves at least two unprovoked seizures occurring more than 24 h apart [28]. Although the etiology of epilepsy is still unclear, it has been postulated that epilepsy involves diverse biological pathways and mechanisms [29]. It is thought that this disorder results from the imbalance of neurotransmitters, specifically glutamate and γ-aminobutyric acid (GABA) [30,31]. Progression of epilepsy results when glutamate concentration is high, causing an increase in Ca^2+^ uptake and generation of frequent depolarization in neurons [32]. GABA, contrarily, maintains the balance of neuron excitation and restricts presynaptic potentials through the hyperpolarization of neurons. [33]. GABA_A_ receptors are ligand-gated ion channels that promote rapid inhibition of presynaptic action potentials by accumulating Cl^−^ concentration in neurons [29]. GABA_B_ receptors are G-protein-coupled receptors that function similarly to GABA_A_ receptors but instead induce slow inhibition by increasing K^+^ and decreasing Ca^2+^ flow into neural cells [29]. In recent studies, serotonin neurotransmitters have also shown to play an important role because of their presence on neurons in the brain. Serotonin is also known as 5-hydroxytryptamine (5-HT) and is able to limit neuronal excitability by activities of various 5-HT subtypes [29].

**Figure 1:**
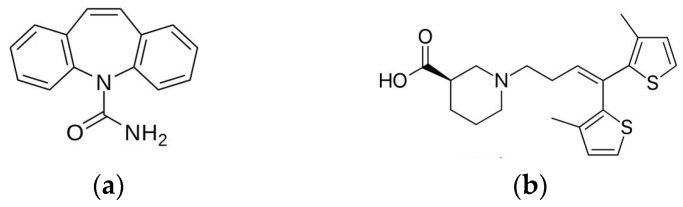
Chemical structures of (**a**) carbamazepine and (**b**) tiagabine.

One of the widely used drugs for the management of epilepsy is carbamazepine (Figure 1a). Carbamazepine (CBZ) has a molecular weight 236.27 g/mol and is a first-choice antiepileptic drug due to few adverse effects [34]. Sold under brand name Tegretol^®^, the drug is a dibenzazepine tricyclic compound that functions as a sodium channel blocker [35,36]. CBZ acts postsynaptically by blocking voltage-gated sodium channels, resulting in the restriction of repetitive firing of action potentials [34,37]. Recent studies have shown that CBZ inhibits the *N*-methyl-d-aspartate (NMDA) subtype of glutamate receptor while pumping K^+^ out of neurons and stabilizing inactive Na^+^ channels with high binding rate constant [38,39]. Inhibition of NMDA receptors is the main antiepileptic property of CBZ because cations are not able to enter neurons. The restriction of cation entry prevents neural depolarization of action potentials.

Conventionally, transdermal drug delivery research is usually restricted to low-dose, potent compounds with optimal physicochemical properties [40]. Recently, though, more research laboratories are devoting considerable time and effort to the development of transdermal drug delivery systems for high dose compounds such as ibuprofen sodium [41] and carbamazepine prodrugs [42]. The rationale for this development is that most medications are high dose, low potency compounds [43]. It is well established that many drugs require high daily doses to obtain therapeutic plasma concentrations [43]. There is therefore an unmet need for enhancement techniques with the potential of delivering high dose medications across the skin. This was the scientific basis upon which we embarked on the transdermal delivery of carbamazepine.

Another drug used for the management of epilepsy is tiagabine (Figure 1b). The drug finds clinical application as an add-on therapy for patients over the age of 12 with partial epilepsy that has not been successfully managed by other antiepileptic drugs [44]. The drug has a molecular weight 412.01 g/mol and acts by inhibiting presynaptic uptake of GABA neurotransmitters in the brain at the synaptic cleft [45]. Limiting uptake of GABA at the presynaptic neurons results in more GABA present for receptor binding to postsynaptic neural cells. In fact, GABA unlocks chloride channels on neurons, allowing hyperpolarization of neuronal cell membranes [46]. The use of tiagabine has two benefits: higher physiological specificity where only released GABA is affected, and reduction of possible side effects [44]. Temporary inhibition of GABA neurotransmitters is currently the only known mechanism of action of tiagabine [39].

Antiepileptic drugs are generally available as tablets for oral administration, but this can pose problems for patients who are unable to consume medications orally [47]. These patients may have difficulty in swallowing, may be in coma or may have gastrointestinal discomfort [22]. Patient compliance may be a problem for those who cannot tolerate the taste of oral medications. Additionally, polypharmacy can be an issue, especially in the elderly population, causing noncompliance and forgetfulness due to the requirement of frequent dosing. Transdermal drug delivery is useful in such cases [17,48,49].

The purpose of our research was to investigate the effect of microneedle rollers on the percutaneous penetration of two antiepileptic drugs, carbamazepine and tiagabine hydrochloride, across pig skin. Pig skin is inexpensive and can be used as a surrogate for human skin [50]. According to Flaten et al., stratum corneum thickness, viable epidermis, and hair follicle and density parameters between porcine and human skin are within the ranges of one another[50]. The use of human skin is ethically challenging despite being considered as the “golden standard” in drug diffusion studies [51,52]. Pig ear skin was porated with stainless steel microneedle rollers and the transdermal flux of drugs was monitored.

## 2. Materials and Methods

### 2.1. Materials

Phosphate buffer saline (PBS, 0.1 M, pH 7.4), ethanol, propylparaben, tiagabine hydrochloride, and carbamazepine were bought from Sigma Aldrich Co. (St. Louis, MO, USA). Tiagabine hydrochloride and carbamazepine were reconstituted in only PBS and PBS/ethanol mixture (20% and 30% ethanol), respectively. Distilled ionized water was obtained from NanoPure Infinity Ultrapure water purification system (Barnstead, Dubuque, IA, USA). The length and density of the microneedles on the microneedle roller were 500 and 192 μm, respectively. The rollers were purchased from Pearl Enterprises LLC (Lakewood, NJ, USA).

### 2.2. Methods

#### 2.2.1. Skin Preparation

Pig ears were purchased from Pel-Freez Arkansas LLC (Rogers, AR, USA). Upon arrival, ears were shaved with an electric hair clipper. Full-thickness skins were obtained by using a scalpel to remove subcutaneous fat from ear cartilage. An electric dermatome (Nouvag^®^, Goldach, Switzerland) was used to prepare split-thickness skins. The thickness of the skin samples was determined with the Digimatic micrometer (Mitutoyo, Tokyo, Japan). Skin membranes were wrapped in aluminum foil and stored at −20 °C until ready for use. Experiments were approved by the Institutional Animal Care and Use Committee (IACUC) and Institutional Biosafety Committee (IBC) of Touro University, Mare Island-Vallejo, CA, USA.

#### 2.2.2. Diffusion Studies

Microchannels were created on porcine skins using solid microneedle rollers and skin samples were then clamped to the diffusion cells. The roller was applied to the pig ear skin 15 times (five times in the vertical, horizontal and diagonal directions). The roller was applied with a force of 5 kg (measured with a weighing balance) for approximately 1 min per application. Three microneedle-treated split-thickness skin (STS) samples were used for the experiment while three untreated skin samples served as controls. Diffusion experiments for tiagabine hydrochloride and carbamazepine were replicated six times (*n* = 6). Each experiment was carried out using 1 mL of either tiagabine hydrochloride (~5 mg/mL) or carbamazepine (~1 mg/mL). The compositions of formulations used in this study are shown in Table 1. The drug was placed on porcine skin. The pig skin was sandwiched between the donor and receptor compartments of Franz diffusion cells. The donor compartment and covered with parafilm and aluminum foil to reduce evaporation. The sampling ports were also sealed. Aliquots of 1 mL were taken using 1 mL syringes from the sampling port every 2 h for 12 h. Extractions were stored in 2 mL amber vials from Agilent Technologies (Agilent, Santa Clara, CA, USA) and stored at −8 °C until shipment to the University of Idaho for analysis. Receptor chambers were replenished after each extraction with 1 mL of fresh, pre-warmed PBS maintained at 37 °C.

We carried out in vitro diffusion studies with six vertical Franz Diffusion cells (PermeGear, Hellertown, PA, USA). Each cell has donor compartments and receptor compartments. In each cell, there is a magnetic stirrer, sampling port, and a water jacket maintained at a 37 °C model human body temperature. The receptor compartment has a diffusion area of 1.77 cm^2^ and the volume is 12 mL. The receptor compartment was filled with PBS, and we utilized high-vacuum grease (Dow Corning, Midland, MA, USA) and a metal clamp to prevent loss of the drug solution through lateral diffusion.

#### 2.2.3. HPLC/DAD/TOF-MS Analysis of Pharmaceuticals

HPLC analysis was performed using an Agilent 1200 Series HPLC system with a diode array detection (DAD) system coupled to an Agilent G1969A TOF-MS system equipped with an ESI source (Agilent, Santa Clara, CA, USA). The chromatographic analysis of tiagabine hydrochloride and carbamazepine was performed using a Zorbax Eclipse Plus C_18_, 100 mm × 2.1 mm, 3.5 µm column (Agilent, Santa Clara, CA, USA) maintained at 30° C. The injection volume was 5 µL. The mobile phase comprised of 0.1% formic acid in water (solvent A) and 0.1% formic acid in methanol (solvent B). We first used a linear gradient from 15% to 95% B in 7 min, and then isocratic elution was carried out at 95% B for 2 min. The final equilibration was conducted at 15% B for 5 min. We diverted the flow from the mass spectrometer for the first 3 min of the analysis, in order to prevent MS contamination and ion suppression. The flow rate was 0.3 mL·min^−1^.

Electrospray ionization was operated in the positive mode. The absolute values for electrospray ionization potential and collision-induced dissociation potential were 3500 and 175 V, respectively. Gas temperature was 350 °C, drying gas (N_2_) flow rate was 12 L·min^−1^, and nebulizer pressure was 2.4 × 10^5^ Pa. The analyses were conducted in a profile mode with an m/z ranged from 90 to 500 amu. Quantification was performed in the reconstructed ion current mode using *m*/*z* of 237.10 (carbamazepine), 376.14 (tiagabine), and 181.09 (propylparaben, used as internal standard).

#### 2.2.4. Microchannel Visualization

Visualization of pores created by the microneedle roller was carried out with a Nikon SMZ-745T dissecting microscope/zoom stereomicroscope (Nikon Instruments Inc., Melville, NY, USA). Porcine skin samples were treated with a 500 μm microneedle roller for 5 s and then stained for 1 min with either Fast Green FCF (Sigma Aldrich Co., St. Louis, MO, USA) or Alexa Fluor^®^ 488 (Life Technologies, Eugene, OR, USA). Skin samples were then rinsed three times with 1 mL portions of normal saline to remove excess dye and then blotted dry with Kimwipes (Kimberly-Clark Professional, Roswell, GA, USA). Control skin samples were treated in the same manner but without the use of microneedles. Images of both untreated and microneedle-treated skins were obtained using a Nikon SMZ-745T dissecting microscope/zoom stereomicroscope equipped with a microscope camera port and built-in 0.55x C-mount adapter for direct mounting of Nikon DS Series Digital Camera (Nikon Instruments Inc., Melville, NY, USA).

#### 2.2.5. Data Analysis

The average cumulative amount of drug permeation through porcine skin was plotted as a function of time. Steady-state flux was calculated from the linear portion of the average cumulative amount versus time curve. Six replicates were utilized for each drug. Sampling effects were taken into consideration when calculating drug concentration by using Equation (1) as suggested by Hayton and coworkers [53]:

(1) $$C_{n}^{1}=C_{n}\left(\frac{V_{T}}{V_{T-V_{S}}}\right)\left(\frac{C_{n-1}^{1}}{C_{n-1}}\right)$$

In the Hayton–Chen equation, $C_{n}^{1}$ is the corrected concentration and $C_{n}$ is the measured concentration in the n^th^ sample. $V_{T}$ represents the total volume of the receiver fluid (12 mL) and $V_{S}$ is the volume of sample withdrawn from the receiver fluid (1 mL). $C_{n-1}^{1}$ and $C_{n-1}$ are the corrected and measured concentration, respectively, in the (*n* − 1)^th^ sample.

#### 2.2.6. Statistical Analysis

Statistical analysis was performed using GraphPad Prism 7 (GraphPad Software, Inc., La Jolla, CA, USA). The Mann–Whitney Rank Sum test was carried out to determine statistical significance. A *p*-value of less than 0.05 was considered statistically significant. The average of replicate measurements (*n* = 6) with corresponding standard deviation was used to plot the graphs.

## 3. Results

### 3.1. Microchannel Visualization

**Figure 2:**
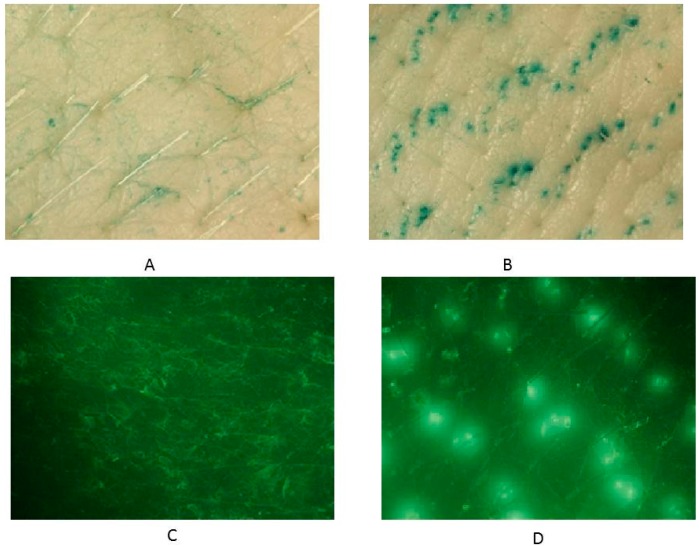
Microchannel visualization using Fast Green FCF on (**A**) untreated skin and (**B**) microneedle-treated skin; Alexa Fluor^®^ 488 on (**C**) untreated skin and (**D**) microneedle-treated skin using 7.5× magnification.

Microneedles were used to disrupt the stratum corneum barrier and enhance transdermal drug delivery. Figure 2 shows the pores created by the microneedle roller on full-thickness skin compared to untreated porcine skin after staining. The pores (Figure 2B,D) were identified as the openings filled with either Fast Green FCF (Figure 2B) or Alexa Fluor^®^ 488 (Figure 2D). Fluorescence was viewed using the NIGHTSEA™ add-on light and filter set with royal blue color light head (Electron Microscopy Sciences, Hatfield, PA, USA). The skin surrounding the microchannels remained intact without any tear in the stratum corneum. Figure 2B,D show the symmetrically aligned pores that imitate the pattern of the needles on the roller itself. Untreated porcine skins were utilized as control samples (Figure 2A,C) to confirm that the microchannel patterns on the skin surface resulted from microneedle application. Very few reports have examined the kinetics of micropore closure following microneedle application [69]. However, it has been documented that following the use of microneedles in humans, pores may close as quickly as 15 min or as long as several hours [69].

### 3.2. In Vitro Transdermal Drug Delivery

**Figure 3:**
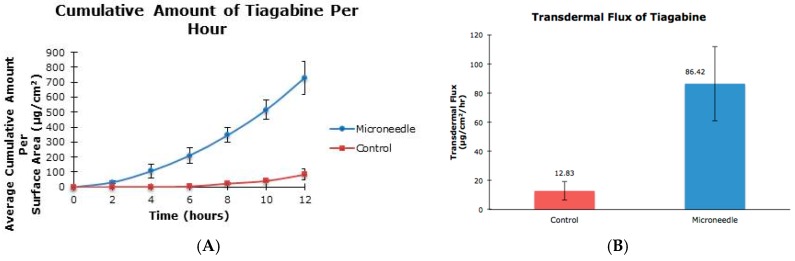
In vitro cumulative amount versus time curve (**A**) and transdermal flux (**B**) of tiagabine hydrochloride across untreated and microneedle-treated (500 μm needle length) porcine ear skin over 12 h.

**Figure 4:**
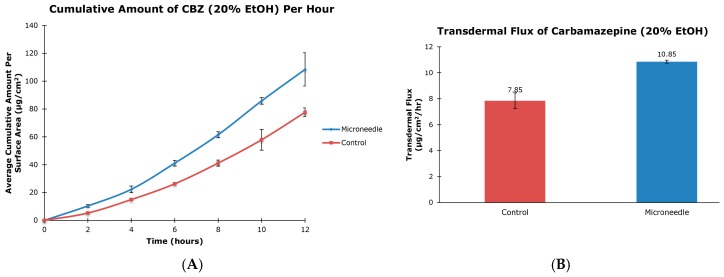
In vitro cumulative amount versus time curve (**A**) and transdermal flux (**B**) of carbamazepine, reconstituted in 20% ethanol, across untreated and microneedle-treated (500 μm needle length) porcine ear skin over 12 h.

**Figure 5:**
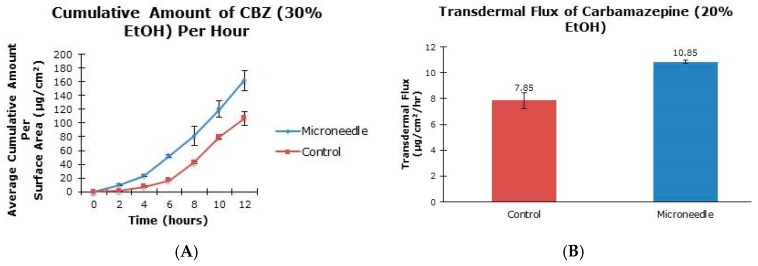
In vitro cumulative amount versus time curve (**A**) and transdermal flux (**B**) of carbamazepine in 30% ethanol across untreated and microneedle-treated (500 μm needle length) porcine ear skin over 12 h.

The cumulative amounts per hour of both tiagabine hydrochloride (Figure 3A) and carbamazepine in 20% and 30% ethanol (Figure 4A and Figure 5A, respectively) are higher after microneedle application when compared to untreated skin. In vitro percutaneous flux of tiagabine hydrochloride increased from 12.83 ± 6.30 μg·cm^2^·h across untreated skin to 86.42 ± 25.66 μg·cm^2^·h across microneedle-treated skin, as shown in Figure 3B. Although we did not specifically measure resistance in our experiments, several reports in the literature indicate that transcutaneous flux increases can be correlated with decreased skin resistance. Carbamazepine reconstituted in 20% ethanol (Figure 4B) showed a rise in flux from 7.85 ± 0.60 μg·cm^2^·h to 10.85 ± 0.11 μg·cm^2^·h. Lastly, the total flux for carbamazepine dissolved in 30% ethanol across microneedle-treated porcine skin (Figure 5B) was 36.73 ± 1.83 μg·cm^2^·h compared to 30.74 ± 1.32 μg·cm^2^·h across untreated skin. Transdermal fluxes of tiagabine hydrochloride and carbamazepine in 20% and 30% ethanol for untreated and microneedle-treated skins are summarized in Table 2. Remarkably, there was no flux increase for CBZ. This is not surprising, as there are reports in the literature showing that the flux of poorly soluble and hydrophobic compounds may not always be enhanced. For example, Vitorino and coworkers did not observe significant transdermal flux enhancement of simvastatin or olanzapine after application of nanostructured lipid carriers [68].

## 4. Discussion

Tiagabine hydrochloride has a molecular weight of 412.01 Daltons. The partition coefficient of the compound is 2.6 and the melting point is 193–195 °C. With an aqueous solubility of 10 mg/mL, tiagabine hydrochloride readily dissolves in phosphate buffer saline, pH 7.4. It has been documented that the therapeutic drug concentration is 20–200 ng/mL, and patients are advised to take less than 56 mg/day [54,55].

The compound is instantaneously absorbed in the gastrointestinal (GI) tract around 30 to 90 min after oral administration with 90% bioavailability and elimination half-life of 5 to 9 h [44]. After drug administration, tiagabine can readily cross the blood–brain barrier and mainly localizes in the cerebral cortex and hippocampus [39]. Passing through the blood–brain barrier is made possible since tiagabine is an analogue of nipecotic acid that is attached to a lipophilic anchor [39]. Volume of distribution is 1 L/kg and less than 1% is unchanged when excreted as urine [42].

Carbamazepine has a partition coefficient of 2.7 ± 0.3 and a melting point of 189 ± 0.71 °C with aqueous solubility of 440.6 ± 20.6 μg/mL at 32 °C in phosphate buffer saline of pH 7.4 [42]. The compound’s molecular weight is 236.27 Daltons. Carbamazepine has a low aqueous solubility and, therefore, was reconstituted in a mixture of ethanol and phosphate buffer saline. Even though the use of microneedles does not always lead to transdermal flux enhancement [56], Gomaa et al. have observed that microneedle-assisted transdermal delivery of drugs with unfavorable skin absorption properties has the potential of bringing to clinical practice more effective and safer products [57]. It was important to evaluate in the present study, if indeed microneedles will facilitate the percutaneous penetration of CBZ.

The oral dose of CBZ in adult patients ranges from 400 to 2000 mg/day, with 400 mg being a recommended dose for most patients [58,59]. Dosages are generally adjusted according to age and severity of epilepsy to obtain optimal therapeutic doses. The therapeutic drug concentration of CBZ ranges between 4 and 12 mg/L [60]. Bioavailability is approximately 75%–85% and half-life is 10–20 h [59]. Volume of distribution is 0.5–2 L/kg and clearance 0.112 ± 0.0147 L/h/kg [61,62]. Interestingly, more exposure to CBZ leads to faster half-life and clearance. Because CBZ is lipid soluble, it slowly breaks down in the gastrointestinal (GI) fluid. CBZ is metabolized in the liver, forming several metabolites including carbamazepine-10,11-epoxide [35,63]. Only 1% of CBZ is excreted in urine unchanged [59].

Typically, the compounds delivered by microneedles have high potency, meaning only a low dose is required to achieve a therapeutic effect [41]. From the clinical standpoint, the majority of commercially available active pharmaceutical ingredients are not low dose high potency molecules. In the contemporary clinical setting, many medications require daily oral doses of several hundred milligrams [41]. The percutaneous route of drug administration has conventionally been used for relatively lipophilic, low molecular weight and potent medications. Because most drug molecules do not have these optimal properties, commercially available transdermal patches have been developed from about 20 active pharmaceutical ingredients [41]. Indeed, the transdermal drug delivery route has proved efficacious for a number of medications used in the management of neurological disorders. For instance, rotigotine is delivered in therapeutic concentration from the transdermal drug delivery system (Neupro^®^) [25]. In addition, the transdermal delivery route bypasses and avoids the substantial hepatic first pass metabolism observed with the oral medication. To deliver high dose medications, some research laboratories have developed specific reservoir-based microneedle systems. One of these systems was reported by McCrudden and coworkers [41].

Another interesting consideration is that several innovative microneedle systems are being developed daily. However, it does not matter how sophisticated these systems are if they do not deliver therapeutic doses of medications. There are also several mathematical models to describe and/or predict the skin permeation of drugs from microneedles [64]. However, only experiments can validate transcutaneous flux values of medications. We took into consideration the above-mentioned facts when we chose two different antiepileptic agents, tiagabine hydrochloride and carbamazepine, for our research project. The results of this project will guide our future development of microneedle systems.

In our study, we showed that the application of microneedle rollers led to increased flux values of tiagabine hydrochloride. To our knowledge, this is the first time that microneedle-assisted delivery of tiagabine hydrochloride has been investigated. However, it is noted that developments of a transdermal patch for tiagabine hydrochloride is currently in its preclinical stage [65]. A US patent (US 5750140 A) was also filed in 1998 for this transdermal drug delivery system [66]. Percutaneous penetration of carbamazepine has been previously studied by Fourie et al. In their study, the authors showed that the steady state flux value for carbamazepine was 3.29 ± 0.64 μg·cm^2^·h across untreated epidermal tissue [42]. Our present study recorded transdermal flux values across untreated porcine skin as 7.85 ± 0.60 and 30.74 ± 1.32 μg·cm^2^·h for carbamazepine reconstituted in 20% and 30% ethanol, respectively. Numerous factors can cause a lack of transdermal flux enhancement, which we observed in this project. The physicochemical properties of a drug can influence the rate of transdermal drug penetration. Hoang et al. alluded to the fact that low diffusion coefficient can lead to low flux values [24].

Carbamazepine is a high dose molecule and although the transdermal drug delivery potential of this drug has been investigated, our knowledge of the factors influencing percutaneous penetration of this compound is still poor. Fourie and coworkers, for instance, used full-thickness skin (FTS) obtained from the abdomen of female patients to study the transcutaneous penetration of CBZ [42]. The epidermal layers were then separated from excess fat layers by immersing skin samples in 60 °C water prior to use. The authors prepared carbamazepine solutions in phosphate buffer saline (PBS) without addition of ethanol. For our study, we prepared split-thickness (STS) porcine ear skins using a dermatome to achieve skin thicknesses of 757 ± 80 μm and 857 ± 90 μm for 20% and 30% ethanol, respectively. Porcine skin is an inexpensive and ideal substitute for human skin due to many similarities of the skin layers [50]. According to Flaten et al., stratum corneum thickness, viable epidermis, and hair follicle and density parameters between porcine and human skin are within the ranges of one another. The use of human skin is generally not feasible and unethical, despite being considered as the “golden standard” in drug diffusion studies [50,67]. We reconstituted carbamazepine in an ethanol and PBS mixture to increase the drug’s solubility. Ethanol diffuses through the skin and improves the permeability of the SC by interfering with the lipid matrix [51]. In addition, 20% and 30% ethanol ratios were chosen because ethanol concentrations greater than 40% may affect the SC barrier function due to possible back-diffusion and enzymatic degradation [50].

It is well established that the use of microneedles [52] and other penetration enhancers (physical and chemical) can lead to transdermal drug delivery flux enhancement. However, there are instances where flux enhancement is not demonstrated [68]. As an example, Vitorino et al. did not observe significant transdermal flux enhancement of simvastatin or olanzapine after application of nanostructured lipid carriers [68]. The authors attributed low permeation to poor solubility and the hydrophobicity of the investigated compounds. Low solubility may also be responsible for the low penetration of CBZ observed in our study. Even though ethanol was effective in increasing the flux of simvastatin and olanzapine in the study by Vitorino and coworkers [68], no statistically significant enhancement effect was observed in our study. This may be attributed to the fact that ethanol has a well-known carry-in effect, which is efficient initially but does not persist due to quick disappearance of ethanol by evaporation and fast diffusion.

## 5. Conclusions

The use of solid microneedles has a significant influence of the transdermal delivery of tiagabine hydrochloride. Our results demonstrated a statistically significant enhancement in the flux of tiagabine hydrochloride, showing a 6.74-fold increase after microneedle application. Conversely, we determined that the differences in the transcutaneous flux of carbamazepine across untreated and microneedle-treated skins were not statistically significant.

## Figures and Tables

**Table 1:** Formulations of tiagabine and carbamazepine used in the experiments.

Name of Solution	Drug Concentration (mg/mL)	Solvent(s)	Solvent Volume (mL)
Tiagabine hydrochloride	5	Phosphate buffered saline (PBS)	7 mL PBS
Carbamazepine in 20% Ethanol	1	PBS + Ethanol	5.6 mL PBS + 1.4 mL Ethanol
Carbamazepine in 30% Ethanol	1	PBS + Ethanol	4.9 mL PBS + 2.1 mL Ethanol

**Table 2:** Transdermal flux (μg/cm^2^/h ± SD) of tiagabine hydrochloride and carbamazepine in 20% and 30% ethanol following treatment with a 500 μm microneedle roller. Passive flux values served as controls (*n* = 6).

Name of Solution	Passive (µg/cm^2^/h)	Microneedle (µg/cm^2^/h)	Flux Increase	*p*-Value
Tiagabine hydrochloride	12.83 ± 6.30	86.42 ± 25.66	6.74	0.039
Carbamazepine in 20% ethanol	7.85 ± 0.60	10.85 ± 0.11	1.38	0.138
Carbamazepine in 30% ethanol	30.74 ± 1.32	36.73 ± 1.83	1.19	0.219
